# Reduced duration mismatch negativity in adolescents with psychotic symptoms: further evidence for mismatch negativity as a possible biomarker for vulnerability to psychosis

**DOI:** 10.1186/1471-244X-13-45

**Published:** 2013-02-02

**Authors:** Jennifer R Murphy, Caroline Rawdon, Ian Kelleher, Deirdre Twomey, Patrick S Markey, Mary Cannon, Richard AP Roche

**Affiliations:** 1 Department of Psychology, National University of Ireland, Maynooth, Co, Kildare, Ireland; 2 Department of Psychiatry, Royal College of Surgeons in Ireland, Dublin, Ireland; 3 Department of Psychiatry, Education and Research Centre, Beaumont Hospital, Dublin, Ireland

**Keywords:** MMN, Mismatch negativity, Psychotic symptoms, Schizophrenia, Psychosis

## Abstract

**Background:**

Deficits in the mismatch negativity (MMN) and P3a components are the most reliable and robust findings in schizophrenia. These abnormalities have also been recently documented in individuals clinically at risk for psychosis, indicating that the MMN may be a potential biomarker for psychosis. However, the at risk samples included in MMN studies are characterised by pre-existing clinical symptomatology and significant functional decline which are related to MMN amplitude. These factors may be potential confounds in determining whether deficient MMN is present prior to clinical manifestation of the disorder. Therefore, investigating the MMN in the extended psychosis phenotype comprising adolescents with psychotic symptoms from the general population may provide important information on whether abnormal MMN is apparent in the earliest stages of risk.

**Methods:**

Thirty six adolescents completed a duration deviant MMN task. Fourteen adolescents with psychotic symptoms comprised the at risk group and 22 with no psychotic symptoms comprised the Controls. The task consisted of 85% standard tones (25 ms) and 15% deviant tones (50 ms). The groups were compared on MMN and P3a amplitude and latency across frontocentral and temporal electrodes.

**Results:**

Adolescents with psychotic symptoms were characterised by a reduction in MMN amplitude at frontal and temporal regions compared to the controls.

**Conclusions:**

This is the first study to demonstrate impaired auditory discrimination for duration deviant tones in nonclinical adolescents with psychotic symptoms. These findings suggest that MMN amplitude may be a possible biomarker for vulnerability to psychosis.

## Background

Disturbances in sensory information processing are considered to be important pathophysiological mechanisms underlying the development of psychosis [1]. Electrophysiological markers of these impairments have been proposed as potential biomarkers for schizophrenia. In particular, reduced amplitude of the duration deviant mismatch negativity (MMN) component is one of the most robust and reliable findings in schizophrenia [2].

Mismatch negativity is an automatic neurophysiological response to a deviant auditory stimulus that is interspersed with a series of standard auditory stimuli [3,4]. A deviant stimulus may differ on a range of auditory dimensions, such as frequency (pitch), duration, intensity or location [5,6]. The MMN waveform is an early negative ERP that is maximal over frontocentral areas of the scalp and peaks between 100-200 ms. MMN tasks are usually passive and undemanding, and the MMN can be elicited without the participant consciously attending to the auditory stimuli [7,8]. MMN is therefore an index of the brain’s ability to extract potentially relevant information from an irrelevant background [9]. Multiple MMN generators have been reported in the auditory cortices and in the prefrontal cortex as part of a distributed fronto-temporal network subserving change detection and attentional switching [10-13].

Studies examining the MMN in chronic patients with schizophrenia [2,14,15], first episode patients [9,16], prodromal individuals [1,17,18] and those genetically at risk [19-23] have reported reductions in MMN amplitude, particularly to duration deviants. Bodatsch et al. [1] found that reduced duration MMN amplitude distinguished between at risk individuals who converted to psychosis from those who did not. Shin et al. [18] also reported the presence of duration MMN deficits in at risk individuals, regardless of conversion. However, not all studies have found deficient MMN as normal mismatch negativity has been reported in unaffected family relatives of patients with schizophrenia [24,25] and also in first episode patients [25,26]. MMN latency findings are inconsistent in schizophrenia studies as some have found delayed latency [27], whereas others have reported shortened [28] or normal MMN latency [29].

At frontocentral electrodes, the MMN wave is often followed by a positive-going ERP component peaking between 250-300 ms [7]. This early P300 component (i.e. P3a) is an automatic process which is thought to reflect an involuntary attention orienting response to a novel stimulus [7,19,30,31]. P3a amplitude in MMN studies is reduced in patients with schizophrenia and also in first episode psychosis [9].

Epidemiological research over the past decade has demonstrated that psychotic symptoms are far more prevalent in the population than actual psychotic disorder [32]. A recent meta analysis of population studies showed that 17% of children and 7.5% of adolescents report psychotic symptoms [33]. These young people have been shown to share a wide range of risk factors with schizophrenia patients [34] and longitudinal research has demonstrated an increased risk of psychotic disorder in adulthood [35,36]. For these reasons, this population has been considered to represent an ‘extended psychosis phenotype’ [37] and researchers have advocated studying aetiological and risk factors for psychosis in this wider phenotype [38,39] including underlying genetic and biological factors [40], as opposed to the ‘narrow’ clinical phenotype of schizophrenia. This approach allows investigation of the earliest risk factors associated with psychosis while excluding potential confounds such as disease chronicity, medication effects and other illness related factors.

While there has been a great deal of clinical research on the extended psychosis phenotype to date, there has been limited neurobiological research on this population. Jacobson et al. [41] demonstrated a fronto-temporal dysfunction in adolescents with psychotic-like experiences compared to a control group and Laurens et al. [42], meanwhile, found electrophysiological evidence for disrupted error monitoring in adolescents with psychotic-like experiences from a community sample. This is the first study to investigate the MMN component in adolescents with psychotic symptoms in a community based setting. As the MMN possesses a relatively high specificity for schizophrenia, the investigation of this component in at risk individuals may increase our understanding of the sensitivity of the MMN as an indicator of vulnerability to psychosis.

## Methods

### Participants

Participants were recruited from the larger Adolescent Brain Development Study (see Kelleher et al. [43] for details). In brief, adolescents aged 11-13 years were recruited from primary (junior) schools in counties Kildare and Dublin and invited to compete a symptom survey in the classroom and attend for clinical interview. Written consent was obtained from both the participant and their parent or guardian and assent was obtained from the adolescents before the study commenced. Following the clinical interview adolescents with psychotic symptoms and control adolescents were sampled from the population-based study and invited to attend National University of Ireland, Maynooth for the Mismatch Negativity Study.

Thirty six adolescents aged 11-13 years agreed to take part in this EEG study comprising 14 with psychotic symptoms (the At-Risk group) and 22 without psychotic symptoms (the control group). All participants had normal hearing and had no previous brain injuries or neurological disorders. No participants were on any medication at the time of testing or had any family history of psychotic illness. Ethics approval was obtained from the ethics committees in the National University of Ireland, Maynooth and in Beaumont Hospital, Dublin 9 (ref no. 06/21).

### Assessment of psychotic symptoms

Psychotic symptoms were assessed using the Schedule for Affective Disorders and Schizophrenia for School Aged Children, Present and Lifetime versions (K-SADS-PL) [43]. The K-SADS-PL is designed to measure the severity of symptomatology reported by children and adolescents, and to assess current and past episodes of psychopathology according to DSM-III-R and DSM-IV criteria. Children and parents were interviewed separately, both answering the same questions about the child. The psychosis section of the K-SADS was used to assess the participants’ psychotic symptoms (hallucinations and delusions) [44]. All interviewers recorded extensive notes of potential psychotic phenomena in this section of the interview and a clinical consensus meeting was held following the interviews. Auditory hallucinations that were deemed as clinically significant included voices commenting on behaviour, a voice giving commands, voices conversing, whispering voices and voices at varying volumes where the words cannot clearly be distinguished by the individual. Clinically significant delusions included feelings of being watched, unfounded ideas that others are saying negative things about the individual (which are distinguished from paranoia and self-consciousness), and a belief that ghost is communicating directly with the individual.

### MMN paradigm

Participants were presented with 1200 auditory stimuli, of which 1020 (85%) were standard tones of 1000 Hz presented at 25 ms (5 ms rise/fall) and 180 (15%) deviant tones of 1000 Hz at 50 ms (5 ms rise/fall time). The interstimulus interval was 300 ms. Stimuli were presented through the computer speakers at 80 dB, while participants were asked to ignore the sounds and look at a fixation point located in the centre of the computer screen. The task consisted of three experimental blocks of 400 stimuli each with a break of 5 seconds between each block. No motor response or stimulus evaluation was required for the task.

### ERP recording

EEG data were recorded using silver/silver chloride electrodes (Brain Vision©) at 64 electrode sites positioned according to the extended international 10-20 system of electrode placement. Electrodes were attached to an elastic electrode cap (Easy Cap©) which was fastened with a chin strap. Horizontal eye movements (HEOG) were recorded from electrodes positioned at the outer canthus of each eye. Vertical eye movements (VEOG) were recorded from electrodes located above and below the left eye. The reference electrode was placed on the nasion of the nose. Skin was lightly abraded to maintain an impedance level below 10kΩ. The EEG signal was amplified (Brain Vision©) with a bandpass filter of 0.16-100 Hz and a gain of 1000. EEG data were digitised at a sampling rate of 500. The conversion rate was 2000 Hz per channel and the range was 150 mV. Filters were set at a low cut off of 0.53 Hz and a high cut off of 30 Hz. Recordings were notch filtered offline at 50 Hz.

### ERP data

**Figure 1:**
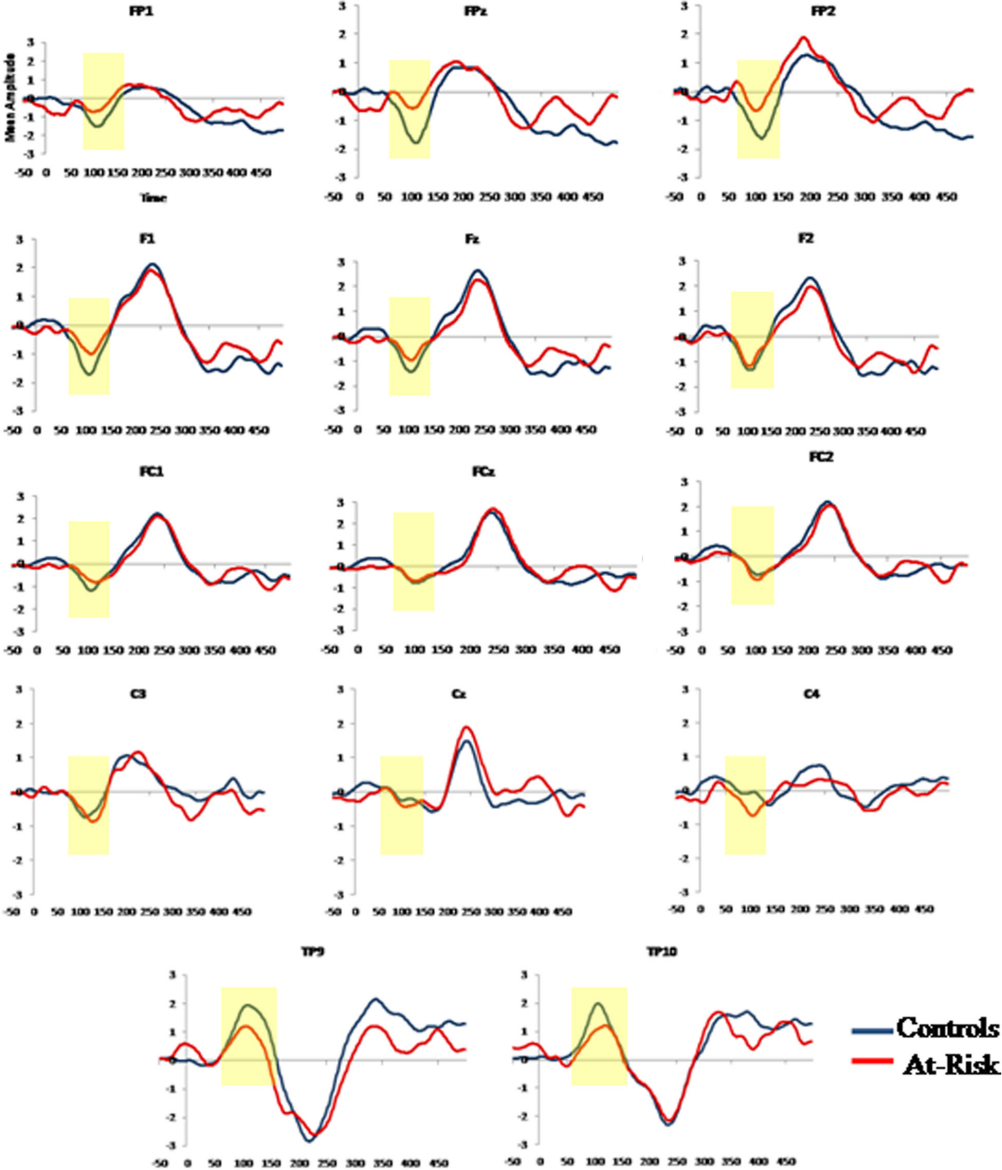
MMN and subsequent P3a activity across frontocentral scalp regions of the controls (blue) and the at risk (red) groups and reversed MMN/P3a polarity at the left and right temporal sites.

Blinks were averaged offline and an EOG automatic artifact rejection algorithm was applied to the data [45,46]. Epochs of 50 ms prestimulus to 500 ms post-stimulus were analysed and baseline corrected to the interval −50 to 0 ms. Mismatch negativity was measured from a difference waveform obtained by subtracting the standard tone ERP waveforms from the deviant tone ERP waveforms. MMN amplitude and latency were measured as the most negative data point within the 80-130 ms latency window, post-stimulus onset. Based on visual inspection of grand averaged waveforms (see Figure 1), MMN amplitude and latency was obtained for each group (Controls, At risk) and condition (Standard, Deviant) over 12 frontocentral electrode sites (FP1, FPz, FP2, F1, Fz, F2, FC1, FCz, FC2, C3, Cz, C4). As the MMN is also detected at mastoid areas with reversed polarity in a nose referenced montage, ERPs were recorded over left and right temporal electrode sites (TP9 and TP10) which were positioned over the mastoid areas (see Table 1). Mean amplitude of the subsequent P3a component was also measured over a latency of 150-290 ms at central electrodes Fz and Cz. The average number of trials (M, SD) accepted for the standard and deviant tones, respectively, were 996.68 (53.67); 171.81 (9.56) for the Controls and 918.78 (136.59); 163.00 (24.05) for the at risk. A 2×2 analysis of variance (ANOVA) verified that there were no group differences in the number of trials accepted [F (1, 34) =2.303, p=.138].

**Table 1:** MMN/P3a mean amplitude (μV) and standard deviation values at frontal and temporal electrodes for each group

##**Electrode**	**Controls**	**At-risk**	**ES *d *(r)**
**Location**	**Mean (SD)**	**Mean (SD)**	**(At-risk vs. controls)**
**FP1**	−1.24 μV (1.11)	−0.60 μV (1.07)	−0.59 (−0.28)
**FPz**	−1.40 μV (1.17)	−0.38 μV (1.40)	−0.79 (−0.37)
**FP2**	−1.24 μV (1.15)	−0.32 μV (1.90)	−0.59 (−0.28)
**F1**	−1.37 μV (0.87)	−0.79 μV (0.81)	−0.69 (−0.33)
**Fz**	−1.09 μV (0.87)	−0.70 μV (1.20)	−0.37 (−0.19)
**F2**	−1.03 μV (1.05)	−0.80 μV (1.30)	−0.19 (−0.10)
**FC1**	−0.91 μV (0.59)	−0.67 μV (0.72)	−0.36 (−0.18)
**FCz**	−0.62 μV (0.73)	−0.52 μV (0.96)	−0.12 (−0.06)
**FC2**	−0.60 μV (0.76)	−0.63 μV (1.09)	0.03 (0.02)
**C3**	−1.56 μV (1.29)	−0.93 μV (0.98)	−0.55 (−0.27)
**Cz**	−0.19 μV (0.66)	−0.22 μV (0.80)	0.04 (0.02)
**C4**	−0.17 μV (0.74)	−0.42 μV (0.92)	0.30 (0.15)
**TP9**	1.69 μV (1.08)	0.93 μV (1.69)	0.54 (0.26)
**TP10**	1.55 μV (1.14)	0.71 μV (1.12)	0.74 (0.35)
**P3a Fz**	1.47 μV (1.00)	1.25 μV (1.34)	0.19 (0.09)
**P3a Cz**	0.29 μV (0.84)	0.81 μV (0.96)	−0.58 (−0.28)

Note: Effect sizes are calculated as the standardised mean differences: ES = (Mean of at-risk group minus the mean of controls)/Pooled SD. ‘μV’ = microvolts.

### Statistical analyses

Statistical analyses were conducted with SPSS for Windows 17.0. Chi-square was employed to compare the at risk and control groups on demographic variables, including age, sex and socioeconomic status (SES). The participants’ socioeconomic status was defined by their parent’s occupations, assessed with the Irish Social Class Scale obtained from the national Central Statistics Office. Mixed factorial ANOVA compared the groups on MMN amplitude and latency. A 2×4 analysis of variance (ANOVA) was conducted where the between groups factor was Group (At risk, Control), and Region (Frontal polar, Frontal; Frontocentral and Central) served as the within groups factor. A subsequent 2×2 ANOVA was also conducted where the between groups factor was Group (at-risk, controls) and the within groups factor was Laterality (left TP9, right TP10). A 2×2 ANOVA compared the groups on P3a amplitude and latency at electrode sites Fz and Cz. An alpha value of p=0.05 was used for statistical significance and Greenhouse Geisser correction was reported when the assumption of sphericity was violated. Bonferroni corrected t-tests were performed to further examine any main and interaction effects. Cohen’s d effect sizes were also calculated to describe the magnitude of any group differences.

## Results

The MMN/P3a waveforms at frontocentral and temporal electrodes for each group are displayed. The at risk and control groups did not differ significantly on demographic variables age, sex, handedness and socioeconomic status (see Table 2). For mean amplitude measures, a 2×4 ANOVA with Group (At risk, Controls) as the between subjects factor with Region (frontal polar, frontal, frontocentral and central) as the within groups factor revealed a main effect of Group [F (1, 34) = 6.235, p = .018]. An interaction effect of Region*Group [F (3, 102) = 5.091, p= .014] was also found. Follow up analyses compared the groups at each region and revealed reduced mean MMN amplitude in the at risk group at the frontal polar region [t (34) = −3.086, p= .004]. A 2×2 ANOVA with Group as the between subjects factor and Laterality (TP9, TP10) as the within groups factor also revealed a significant group difference over temporo-parietal areas [F (1, 34) = 6.323, p=.017]. Follow up independent t-tests revealed reduced MMN amplitude in the at risk group over the right temporo-parietal electrode TP10 in comparison to the Control group [t (34) = 2.660, p=.012].

**Table 2:** The means and standard deviations of demographic variables overall and for each group

**Variable**	**Overall**	**At-risk**	**Controls**	**F & P values P<0.05**
		**(N=14)**	**(N=22)**	
**Age (Mean Years/SD)**	11.48 (.60)	11.57 (.85)	11.41 (.50)	*X*^2^=6.166, df=2, p=.104
**Sex (Male; Female)**	16 M; 20 F	8 M; 6 F	8 M; 14 F	*X*^2^=.773, df=1, p=.379
**Handedness**	35 R; 5 L	13 R; 1 L	19 R; 3 L	*X*^2^=.000, df=1, p=1.000
**SES (N=Professional/Managerial & Other)**	N=34; 6 Other	N=11; 3 Other	N=19; 2 Other	*X*^2^=.255, df=1, p=.613

Note: SES=Socio-economic Status; Number of participant’s parents occupations in Professional/Managerial or Other roles.

For latency measures, a 2×4 ANOVA with Group (At risk, Controls) as the between subjects factor with Region (frontal polar, frontal, frontocentral and central) as the within groups factor was also conducted. A main effect of Region [F (3, 102) = 5.085, p = .006] was found which demonstrated delayed processing over frontal polar regions. The groups did not differ in MMN latency in any region. A 2×2 ANOVA was conducted for Laterality (TP9, TP10) and Group which revealed no significant differences. A 2×2 ANOVA was conducted on P3a mean amplitude comparing Group (At risk, Controls) and Region (Frontal (Fz) and Central (Cz)). A main effect of Region [F (1, 34) = 14.252, p = .001] revealed increased frontal activity compared to central regions. P3a latency was also analysed and a main effect of Region [F (1, 34) = 6.228, p = .018] showed shorter latency in the frontal region in comparison to the central region. The groups did not differ on P3a amplitude or latency.

## Discussion

Adolescents reporting psychotic symptoms from a community sample exhibit deficits in preattentive auditory change detection as indexed by reduced MMN amplitude compared to a control group. This finding is consistent with studies of patients with chronic schizophrenia [2,15,47], first episode patients [9,16] and individuals in the ultra high risk or prodromal stage [1,17,19,48] which have reported reduced MMN amplitude over frontocentral regions. MMN amplitude was also reduced at mastoid electrode sites which is consistent with a previous study in first episode patients [9]. However, this finding is the first to be reported in individuals at risk for psychosis.

The results of the current study support the evidence suggesting that reduced MMN in response to duration deviants may represent a potential biomarker candidate for psychosis. Duration deviant paradigms yield more consistent results and appear to be more sensitive to deficits than frequency deviants. Umbricht and Krljes [49] revealed that the effect size between patients and controls for duration deviants was 40% larger than that for frequency deviants. Deficient MMN in response to a frequency deviant is usually observed in chronic patients and is associated with illness progression and functional status, whereas impaired duration MMN has been proposed as a possible trait marker for schizophrenia [9,50].

P3a amplitude or latency was unaffected in the at-risk group as no group differences were found for this component. Impairments in MMN accompanied by an unaffected P3a may suggest a subtle disruption in the initial automatic detection of change in the auditory environment that is not sufficiently impaired to affect the subsequent attention orienting response. There were no group differences in MMN latency, which supports previous studies reporting a lack of MMN latency differences in chronic patients with schizophrenia compared to controls [29].

Some limitations of this study must be addressed. In view of the relatively small sample size replication of our findings in larger samples of individuals with psychotic symptoms or prodromal syndromes will be required. The lack of longitudinal follow up data limits the ability to draw conclusions regarding the predictive value of reduced MMN amplitude as a marker of vulnerability to psychosis. Follow up studies will elucidate whether the MMN can distinguish between those who develop psychosis or another psychiatric disorder from those who do not. Nonetheless, this study provides important information regarding the utility of the mismatch negativity as a potential biomarker for a broader psychosis phenotype and demonstrates the value of this population in terms of understanding the underlying pathophysiology of psychosis in pre-disease, pre-medication, community based group. The community based approach of the current work also helps to address limitations of clinic based research, including clinically presenting ultra high risk patients, which may be confounded by pre-existing illness related biological changes, with resultant identification of state rather than trait markers for psychosis Belger et al., [51].

## Conclusions

In summary, our results demonstrate that adolescents with psychotic symptoms from the general population are characterised by a reduction in duration MMN amplitude at frontal and temporal areas compared to a control group. These findings are the first to be shown in adolescents from a community sample experiencing nonclinical psychotic symptoms indicating that neurobiological changes, particularly impairments in preattentive auditory processing, are evident long before illness onset during the risk period.

## Competing interests

The authors declare that they have no competing interests.

## Authors’ contributions

JM, CR and IK recruited the participant sample. JM set up and recorded the EEG, analysed the data and wrote the first and final drafts of the manuscript. CR, DT and PSM helped carry out EEG set up and recording. IK conducted clinical interviews with the participants and their parents. RR aided the writing process and the data analysis. MC also helped to write and structure the manuscript. All authors read and approved the final manuscript.

## Pre-publication history

The pre-publication history for this paper can be accessed here:

http://www.biomedcentral.com/1471-244X/13/45/prepub
